# MicroRNAs as prognostic markers and therapeutic targets in gliomas

**DOI:** 10.1016/j.ncrna.2022.07.001

**Published:** 2022-07-06

**Authors:** Albert Sufianov, Sema Begliarzade, Tatiana Ilyasova, Yanchao Liang, Ozal Beylerli

**Affiliations:** a Federal Center of Neurosurgery, Tyumen, Russia; b Department of Neurosurgery, Sechenov First Moscow State Medical University (Sechenov University), Moscow, Russia; c Educational and Scientific Institute of Neurosurgery, Рeoples’ Friendship University of Russia (RUDN University), Moscow, Russia; d Republican Clinical Perinatal Center, Ufa, Republic of Bashkortostan, 450106, Russia; e Department of Internal Diseases, Bashkir State Medical University, Ufa, Republic of Bashkortostan, 450008, Russia; f Department of Neurosurgery, the First Affiliated Hospital of Harbin Medical University, Harbin, 150001, China; g Institute of Brain Science, Harbin Medical University, Harbin, 150001, China

**Keywords:** Brain tumors, Gliomas, microRNAs, Molecular markers, Therapeutic targets

## Abstract

Gliomas are invasive brain tumors characterized by high rates of recurrence and mortality. Glioblastoma (GBM), a grade IV brain tumor, is known for its heterogenicity and its resistance to the current treatment regimen. MicroRNA (miRNAs) are small non-coding sequences of RNA that regulate and influence the expression of multiple genes. The detection of certain types of micro-RNA in tissues and blood serum can be used for diagnosis and prognosis, including the response of a particular patient to therapy. The purpose of this review is to analyze studies and experimental results concerning changes in microRNA expression profiles characteristic of gliomas. Furthermore, miRNAs also contribute to autophagy at multiple stages. In this review, we summarize the functions of miRNAs in GBM pathways linked to dysregulation of cell cycle control, apoptosis and resistance to treatment, and the possible use of miRNAs in clinical settings as treatment and prediction biomarkers.

## 1 Introduction

Central nervous system tumors are diagnosed annually with a frequency of 3.5 per 100,000 people, which accounts for approximately 1.9% of all new cancers and 2.3% of deaths from all cancers in the world [1]. Gliomas are a type of tumor that results from malignant transformation of glial cells, and affecting the brain and spinal cord [2]. According to the WHO classification, according to clinical and pathological criteria, gliomas are divided into stages I-IV. The most dangerous is glioblastoma multiforme - a stage IV tumor. Morphologically, gliomas are subdivided into astrocytomas, oligodendrogliomas, and mixed oligoastrocytomas [3] (see Table 1).

**Table 1:** Major miRs associated with gliomas/glioblastomas.

miR	Research/detection method miR	Expression of miR in glioma	Targets	Commentary	Referenses
let-7	qPCR, miRNA microarrays		Ras, c-Myc, Stat3, cyclin D1	The maturation of let-7 can be blocked by the LIN28 protein, an increased level of which is associated with worse survival. Let-7b may serve as a marker of cisplatin resistance.	[[57], [58], [59], [60]]
miR-7	miRNA microarrays, qPCR		Transcription factors PI3K and Raf-1, EGFR	Since miR-7 is characterized by a very high level of tissue specificity, it can be an ideal therapeutic target. The miR-7 level decreases significantly depending on the WHO stage.	[61,62]
miR-9	qPCR (Taqman), bioinformatics calculations		SPTCH1, OX2	Potential therapeutic target of increased chemosensitivity to temozolomide. Differential expressed in glioma tissues with and without the R132H IDH1 mutation.	[63,64], [61]
miR-10a,b	qPCR		BCL2L11, CDKN2A	They belong to hypoxia-regulated microRNAs.	[61,65], [66,67]
miR-21	Hybridization, qPCR		Multiple targeted pathways including EGFR, PI3K/Akt/mTOR, PTEN	Expression profile differs in oligodendrogliomas and glioblastomas. One of the most hyperactive miRs in gliomas, it blocks apoptosis. It correlates inversely with WHO stage. Mediator of radiation resistance in gliomas.	[6,68]
miR-23b	qPCR		VHL, TK2B	Refers to hypoxia-regulated micro-RNA. Hyporegulation of miR-23b suppresses tumor proliferation across the VHL and inhibition of the b-catenin/Tcf-4 and HIF1a/VEGF signaling pathways.	[61,66]
miR-26a	qPCR		PHB, PTEN	miR-26a inhibits downstream pathways by inhibiting PHB Akt and ERK. PTEN is a specific molecular marker of gliomas, is associated with lifespan, and is a tumor suppressor.	[61,69]
miR-33	RT2 miRNA PCR Array (QIAGEN)		UVRAG, a negative regulator of the Notch pathway	Associated with the worst prognosis for patients.	[70]
miR-34a	qPCR		Notch1, Notch2, cyclin-dependent protein kinase-6 (CDK6), Rictor protein (Akt/mTOR and Wnt pathways), Smad4	Oncosuppressive micro-RNA, the expression level of which has prognostic value for patients with gliomas.	[61,71], [64,72]
miR-125b	qPCR		Hh signaling, CDK6, LIN28, MMP9	miR-125b is hypoactivated in gliomas with increased Gli1 expression compared to other gliomas. The levels of miR-125b and MMP9 were significantly higher in the culture of highly invasive glioma stem cells.	[61,73]
miR-132	qPCR		Smad7	High expression of miR-132 is a marker of the worst prognosis for patients with gliomas.	[61]
miR-146b	qPCR, TCGA Bioinformatics Data Analysis		EGFR, RAF6	Tumor suppressive micro-RNA. The level of miR-146b correlates inversely with the grade of malignancy and positively with the patient's survival.	[74,75]
miR-155	qPCR		HBP-1, MAPK13, MAPK14	Activation of Wnt/b-catenin signaling. Expression of miR-155 allows you to distinguish oligodendroglioma from glioblastoma.	[68,76]
miR-221/222	miRNA microarrays, qPCR		PTPm, MGMT, regulation of the Wnt/b-catenin pathway, RB1, WEE1 (cell cycle inhibitors), APAF1 (pro-apoptotic role), ANXA1, CTCF (transcriptional repressors).	miR-221 and -222 promote glioma growth, at least in part by controlling protein expression of PTPm. miR-221 modulates the mRNA level of 602 genes, which confirms its ability to influence many oncogenic pathways [45]. Silencing of the MGMT gene can also be caused by hypermethylation of CpG islands, which is characteristic of gliomas.	[[77], [78], [79]]

A significant number of different miRs have been found that affect the processes of malignant transformation of cells and the progression of gliomas [4]. The possibility of using miRs as prognostic/diagnostic markers of glioma progression is being actively explored. For example, in the study [5], the profile was identified and validated of 9 micro-RNAs (miR-124a, −10b, −222, −34a, −182, −148a, −145, −370 and −9), which can effectively predict the survival of glioblastoma patients. One of the most hyperactivated miRs in glioma is the antiapoptotic miR-21, whose expression in blood and tissue samples in gliomas is characterized by high specificity and sensitivity as a diagnostic test [6]. It has been shown that those micro-RNAs that are hyporegulated in gliomas compared to normal brain tissues, function as tumor suppressors, directly acting on the oncogenes c-Met, Notch, Bmi-1, EGFR (epidermal growth factor receptor), receptor tyrosine kinases, as well as genes associated with the control of the cell cycle. For example, hyporegulation of miR-205 was found to be an independent predictor of mortality among patients with gliomas, which can serve as a significant prognostic indicator for patients with gliomas of a higher (III – IV) grade of malignancy according to the WHO classification [7]. The activation of miR-497 expression in glioma cells led to a decrease in the expression of the vascular endothelial growth factor (VEGF) and the density of blood vessels, thereby being a negative regulator of angiogenesis. This micro-RNA acts as a favorable prognostic factor: the expression of miR-497 was significantly lower in high-grade gliomas compared to low-grade gliomas [8]. It has been demonstrated that the evolutionarily conserved RNA-binding protein LIN28, capable of blocking the maturation of miR precursors of the let-7 family, is overexpressed in glioblastoma patients with a poor prognosis. A decrease in the level of LIN28 in the cell culture of U251 glioma caused the arrest of the cell cycle in the G1 phase, a slowdown in proliferation, and activation of apoptosis [9]. It was shown that miR let-7 is able to directly regulate the expression of such oncogenes as Ras, c-Myc, as well as the Stat3 transcription factor due to the presence of let-7-binding sites in the 3 ¢ -UTR of these genes; let-7 is inhibited in various types of tumors [10]. Along with this, micro-RNAs with increased expression in tumors can act as oncogenes. Thus, in glioma cells, overregulation of microRNAs such as miR-10b, −130a, −221, −125, −9, −21, −25, and −123 was established in comparison with normal brain tissue. Increased expression of miR-10b and miR-210 in gliomas is associated with unfavorable outcomes [11,12].

This review considers the possibility of using miRNAs as prognostic markers and therapeutic targets in gliomas.

## 2 MICRORNAs as prognostic markers

**Fig. 1:**
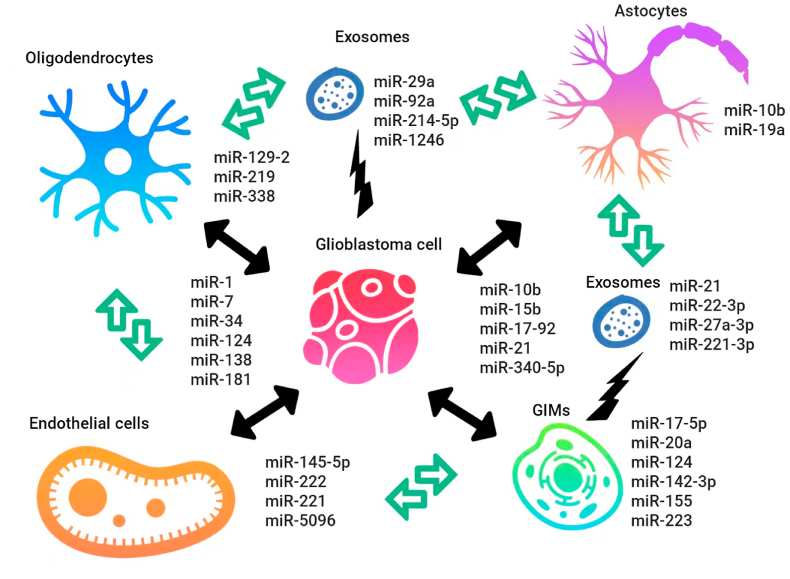
Potential miRNAs that can be used as prognostic markers and therapeutic targets in gliomas.

Many recently published works have confirmed the association of microRNA with chemotherapy and radiation resistance of patients' tumors (Fig. 1) [13].

Radiation resistance, often caused by the spread of CSCs in gliomas, remains one of the main difficulties in the treatment of glioblastoma. In the context of radiation resistance of gliomas, miR-21 is one of the most actively discussed microRNAs. Blocking this microRNA with anti-miR-21 promotes the formation of phosphorylated histone g-H2AX (an indicator of double-stranded DNA breaks) and suppression of phospho-Akt expression, activation of autophagy and apoptosis (including by inhibition of Cdc25A expression) after irradiation in cell lines gliomas [13,14]. MiR-21 also plays a significant role in the chemoresistance of glioblastoma cells: the use of the miR-21 inhibitor together with temozolomide increased glioma CSC apoptosis [15]. Knockdown of miR-135b is capable of leveling the radiation resistance of glioblastoma cells (U87R), and overexpression of miR-135b enhances the radiation resistance of U87 cells [16]. High expression of miR-34a in glioblastoma cells after irradiation with a dose of 60 Gy decreases the expression of p53; thus, the regulation of miR-34a expression can induce apoptosis even in glioblastoma cells that have acquired radiation resistance [17]. Several microRNAs of the let-7 family were overexpressed after irradiation of glioblastoma cells of the M059K line (let-7 suppresses the proliferation of glioma cells), but were overexpressed in the case of other glioblastoma cells of the M059J line. The difference between these two cell cultures is that M059J cells do not possess DNA-dependent protein kinase, which plays a central role in the restoration of double-stranded DNA breaks induced by ionizing radiation. The lack of DNA-dependent protein kinase correlates with the sensitivity of glioblastoma cells to radiation [18]. A significant increase in expression (3–4 times) was found for miR-24-1 and miR-151-5b after irradiation of glioma cell lines using doses commonly used for the treatment of brain tumors - 2 Gy [19]. An increase in the expression of miR-146b-5p in a glioma CSC culture induces apoptosis, cell differentiation and sensitivity to radiation therapy, reduces the viability of glioma CSCs, and the formation of neurospheres. One of the targets of miR-146b-5p is ELAVL1 (ELAV like RNA binding protein 1; also known as HuR), which is associated with a decrease in the level of lincRNA-p21, a regulator of cell proliferation and apoptosis [20]. The level of another microRNA, miR-590-3p, was increased in glioma tissues and radioresistant glioblastoma cells; miR-590-3p was shown to promote radioresistance by directly inhibiting the tumor suppressor LRIG1 (leucine rich repeats and immunoglobulin like domains 1) [21]. It was found that miR-221/222, an increase in the expression level of which was repeatedly noted in gliomas, mediates the restoration of DNA damage in glioblastoma cells, regardless of PTEN, which indicates its potential to be a potential therapeutic target for increasing the radiosensitivity of tumor cells [22]. Hypoxia leads to an increase in the level of HIF2A and miR-210 mRNA in glioma CSCs, while miR-210 knockdown reduced the ability of glioma CSCs to form neurospheres, expression of stem cell markers, and induced differentiation and cell cycle arrest in the G0/G1 phase [23]. In U87MG glioma cells after irradiation, the level of miR-181a decreased, while the transient overexpression of miR-181a significantly increased the response of tumor cells to radiation by inhibiting the antiapoptotic protein Bcl-2 [23]. Along with this, micro-RNAs play an important role in modulating the chemoresistance of glioma cells, including to temozolomide. Thus, it is known that the synthesis of cytochrome CYP3A4, which metabolizes most chemotherapy drugs, including those used in the treatment of gliomas, is increased in brain tumors, and can be suppressed with the participation of certain micro-RNAs, for example, miR-148a, −27b and −125b, which are therefore the targets of reducing the chemoresistance of glioblastomas. These microRNAs suppress nuclear receptors for vitamin D and PRX at the posttranscriptional level [24]. When comparing two glioblastoma CSC lines, CD133 + and CD133–, it was found that CD133 + cells have increased expression of the MDR1 gene (multiple drug resistance gene 1) and miR-9, which activates Shh signaling by decreasing the PTCH1 level. Knockdown of Gli1 and MDR1 by siRNA increases temozolomide-induced cell death [25]. Using HiSeq and bioinformatics approaches, certain long noncoding RNAs have been identified, the level of which differs in patients resistant and sensitive to temozolomide use, with lncRNA MALAT1 (metastasis associated lung adenocarcinoma transcript 1) having the highest discriminating potential [26]. Moreover, high serum MALAT1 expression is associated with an unfavorable response to chemotherapy with temozolomide and poor survival. MALAT1 inhibits miR-203, one of the targets of which is thymidylate synthase; inhibition of MALAT1 may be a further therapeutic strategy for suppressing resistance to temozolomide [26]. It has been shown that overexpression of miR-423-5p confers resistance to temozolomide to glioma cells and enhances the formation of neurospheres [27]; miR-181b has completely opposite effects [28]. It should be noted that the assessment of the expression profile of several micro-RNAs (miRNA-signature), the integration of data on the expression of micro-RNAs, genes, and proteins can reveal valuable biomarkers for the diagnosis and treatment of gliomas, characterized by statistical significance [29]. Thus, 27 differentially expressed micro-RNAs can serve as markers of initiation, progression of glioma, and prognosis; Six of these micro-RNAs were characterized by a greater predictive value (sensitivity, specificity, p) in differentiating glioma cells from non-tumor tissues. From this cluster, miR-15a, −16, −21, −23a, −9 were overexpressed in glioma tissues, while miR-124 was overexpressed. A feature of two microRNAs from this cluster, miR-124 and miR-9, is the fact that they are specifically expressed in the brain [29]. The group of Shi et al. showed that a decrease in the expression of miR-124 in tumor tissue stimulates the growth, angiogenesis, and chemoresistance of glioma; therefore, miR-124 may be a useful diagnostic marker [30]. Circulating miRNAs represent a new class of non-invasive biomarkers for the detection and prognosis of gliomas. Thus, the levels of circulating miR-128, -205, and −451a were lower in the blood serum of patients with gliomas compared to controls or patients with meningioma [[31], [32], [33]]. Analysis using microchips and qPCR, ROC curves showed that the simultaneous determination of the expression level of miR-15b and miR-21 in blood serum helps to differentiate patients with gliomas from patients with other brain lesions (metastases, etc.) with 90% sensitivity and 100% specificity [34]. A meta-analysis of 11 studies that tested the diagnostic informativeness of assessing the expression of various types of micro-RNA in blood and tissue samples in gliomas revealed high values of specificity, sensitivity and AUC (area under the ROC curve) for miR-21; however, the authors of this work note that profiles from a larger number of micro-RNAs will further improve diagnostic accuracy [6]. Indeed, the levels of miR-21, -128, and -342-3p in the blood plasma of patients with glioblastoma and healthy donors discriminated against these samples with high sensitivity and specificity: the level of miR-21 was increased, while miR-128 and miR-342-3p - decreased in glioblastoma, but not in other types of brain tumors. Moreover, the levels of miR-128 and miR-342-3p were positively correlated with the histopathological stage of gliomas [35]. ROC curves for the levels of miR-497 and miR-125b in serum showed high significance in differentiating glioblastomas from tumors of a lower malignancy - the AUC values reached 0.87 and 0.75 for miR-497 and miR-125b, respectively. Moreover, patients with glioblastoma have a significantly reduced expression level of miR-497 and miR-125b than patients with low-grade gliomas [36]. MiR-210 in serum is also a promising diagnostic and prognostic biomarker that can be detected in the peripheral blood of patients with gliomas, since the serum miR-210 level in glioblastoma is approximately 7 times higher than in healthy donors [37]. Another potential biomarker search platform for therapeutic monitoring of patients with gliomas is the analysis of cerebrospinal fluid (CSF) and extracellular vesicles isolated from blood plasma or CSF. Primary brain tumors with a tendency to disseminate can secrete microRNAs with oncogenic properties in the CSF; therefore, the study of microRNAs circulating in the CSF can help in the diagnosis of brain tumors [38]. In extracellular vesicles, CSF is present in high concentrations, which has already been repeatedly noted in connection with glioblastoma, miR-21; in general, CSF exosomes are the main extracellular compartments containing microRNAs [39]. Simultaneous testing of miR-15b and miR-21 expression levels in CSF can differentiate patients with gliomas from healthy people and patients with CNS lymphomas with 90% sensitivity and 100% specificity [40]. SVM (support vector machine, one of the machine learning algorithms) has shown that determining the expression levels of 7 microRNAs in the CSF (miR-10b, −21, −141, −200a, −200b, −200c and −125b) allows to differentiate glioblastomas and brain metastases with high accuracy (91–99%) on the test dataset. Nevertheless, the authors note that the search for additional microRNA biomarkers and subsequent analysis of large cohorts of patients will help to increase the accuracy of the approach [41].

## 3 MICRORNAs as potential therapeutic targets

The principle of therapeutic targeting of micro-RNAs is based, on the one hand, on increasing their expression due to the introduction of synthetic micro-RNAs (micro-RNA mimetics) to inhibit oncogenes; on the other hand, on the inactivation of endogenous micro-RNAs using inhibitors (antagomiRs or anti-micro-RNA) in order to increase the expression of tumor suppressors [42]. Effective inhibition using anti-micro-RNA requires high affinity for targeted micro-RNA, resistance to nuclease degradation, low toxicity, and efficient in vivo delivery. The most widely used anti-micro-RNAs today are modified oligonucleotides carrying the so-called LNA (locked nucleic acid) [43]. For example, the use of an antisense oligonucleotide inhibitor miR-10b led to a decrease in the target level, inhibited the growth and progression of xenograft glioblastomas, while the route of administration (intratumoral injections, intravenous administration, osmotic administration) was unimportant and systemic toxicity was not observed [44]. Nevertheless, the introduction of antisense oligonucleotides, the so-called anti-miRs, into the systemic circulation is complicated by their possible degradation and the presence of the blood-brain barrier (BBB) [45]. To penetrate the BBB, various methods of targeted drug delivery are being developed. Thus, the introduction of anti-let-7 oligonucleotide into xenograft animals with glioblastoma using stereotaxic surgery showed that the most effective methods of administration are intratumoral and intraventricular methods [45]. One of the studies showed that R3V6 peptides are capable of protecting anti-miR-21 oligonucleotides from nucleases, while anti-miR-21 is delivered to the cell much more efficiently than with other modifications [46]. The use of the drug reduced the level of miR-21 and induced apoptosis in glioblastoma cells, indicating that R3V6 can serve as a “carrier” of antisense oligonucleotides [46]. An alternative to antisense oligonucleotides is the so-called “miRNA sponge” (“sponge for micro-RNA”), which contain many binding sites specific to the so-called seed-region of targeted micro-RNAs [43]. On a glioma cell line and a laboratory mouse model, the ability of such a microRNA sponge to inhibit miR-23b, which functions as an oncogene in glioblastoma, was demonstrated, which led to a significant decrease in the level of HIF1a, b-catenin, MMP-2, -9, VEGF, inhibited angiogenesis, migration, and invasion of tumor cells [47]. Synthetic micro-RNA sponges were created on the basis of their natural counterpart - cyclic RNA (circRNA), the role of which in carcinogenesis is just beginning to be studied. For instance, RNA circ-TTBK2 is overregulated in glioma tissues and cell lines, while linear TTBK2 does not change its level [48]. Circ-TTBK2 acts as a sponge for miR-217, the level of which has been reduced in glioma, and whose target is the oncogenic protein HNF1b (HNF1 homeobox beta). Knockdown of circ-TTBK2 together with miR-217 overexpression led to tumor regression in vivo [48]. More recently, a computer pipeline algorithm “UROBORUS” has been developed for circRNA detection based on RNA-seq data [50]. Using this algorithm, more than 476 cyclic RNAs were found, the expression of which differed in the normal brain and in glioma samples [49]. Another analysis [50] revealed 1411 differentially expressed cyclic RNAs in glioblastoma, of which 206 were hyperactivated and 1205 were hypoactivated. At the same time, the level of circBRAF expression in the normal brain was significantly higher relative to the glioma tissue; was lower in patients with high grade (III-IV) gliomas. A high level of circBRAF expression was an independent biomarker of a favorable prognosis for disease-free and overall survival in patients with glioma [50]. Micro-RNA-replacement therapy is aimed at restoring lost function, especially the activity of tumor suppressors, which can be achieved by introducing synthetic micro-RNA mimetics with sequences identical to natural ones [43]. For instance, Transfection of miR-203 mimetics into U251 glioblastoma cells, which showed a decrease in miR-203 levels relative to less malignant gliomas or normal gliomas, led to inhibition of the PLD2 (phospholipase D2) oncogene, the target of miR-203. This suppressed the proliferation and invasion of U251 cells, demonstrating the ability of mimetics to correct microRNA deficiency [51]. Targeted delivery of synthetic microRNA mimetics to glioma cells in vivo and glioma CSCs in culture can be performed by mesenchymal stem cells isolated from bone marrow, adipose tissue, and placenta [52]. The introduction of miR-124 and miR-145 mimetics using mesenchymal stem cells into glioma cells that express very low levels of these microRNAs has shown successful delivery of drugs into cells, where they significantly reduced the luciferase activity of the corresponding target genes miR-124 and miR −145 - SCP-1 and Sox2, and also inhibited glioma cell migration and glioma CSC self-renewal [52]. Another attractive approach in the treatment of gliomas is the use of siRNA, short interfering double-stranded RNA molecules that are potentially capable of specifically inhibiting signaling pathways associated with the development and progression of glioblastomas, for example, the EGFR and b-catenin genes [53]. Genome-wide siRNA screening in glioblastoma showed that 2 siRNAs, which target ubiquitin C and disheveled 2, significantly increase the sensitivity of glioma CSCs to temozolomide [54]. Inhibition of the gene for the transcription factor PATZ1 (POZ/BTB and AT Inhibition of the transcription factor gene PATZ1 (POZ/BTB and AT hook containing zinc finger 1) by siRNA increased the sensitivity of the glioma cell line to apoptotic stimuli [55]. Treatment of glioblastoma cells with a haemagglutinating virus vector HAJ-E containing siRNA targeting the mitotic motor protein KIF11 (kinesin family member 11, also known as EG5) led to the formation of a monopolar division spindle, cell cycle arrest, and apoptosis. Direct injection of HVJ-E + Eg5 siRNA into xenografts subcutaneous/intracranial tumors in all xenograft mice, tumor growth was inhibited [56].

## 4 Conclusions

Expression profiles of certain types of microRNA in gliomas repeat the changes characteristic of other types of malignant neoplasms. Similarly, to genes proper, subdivided according to their effect on tumor pathogenesis on tumor suppressors and oncogenes, by now it is already possible to distinguish classes of microRNAs that also play opposite roles in the pathogenesis and progression of gliomas - oncogenes (for example, miR-21, -96, - 155, etc.) and tumor suppressors (miR-34a, −107, −205, etc.). Studies of microRNA expression profiles have expanded our understanding of the mechanisms of progression of gliomas/glioblastomas, providing valuable information on the pathogenesis of these tumors and potential therapeutic targets (eg, inhibition of anti-apoptotic miR-21) [[81], [82], [83], [80]]. Detection and quantification of a number of micro-RNA species in tissues and blood serum of cancer patients can be of diagnostic and prognostic significance. Micro-RNAs can also be used to predict the response of a particular patient to therapy (for example, miR-338-5p sensitizes glioblastoma cells to the effects of ionizing radiation through the regulation of genes involved in the response to DNA damage) [84,85].

## Funding

None.

## Author contributions

Albert Sufianov and Sema Begliarzade conceptualized and designed the study. All authors participated in the acquisition, analysis and interpretation of the data. Tatiana Ilyasova and Yanchao Liang drafted the manuscript. Ozal Beylerli contributed to critical revisions of the manuscript. All authors agreed on the journal to which the article would be submitted, gave final approval for the version to be published, and agreed to be accountable for all aspects of the work.

## Declaration of competing interest

The authors declare that no conflicts of interest exist.
